# An Effective Treatment for Recurrent Bilateral Facial Palsy in a Flight Attendant: A Case Report

**DOI:** 10.7759/cureus.53517

**Published:** 2024-02-03

**Authors:** Farhana Nur Iman Lokman, Mohd Solahuddin Mohd Kenali, Charlene P Malakun

**Affiliations:** 1 Graduate School of Medicine, Kumpulan Perubatan Johor Healthcare University, Nilai, MYS; 2 Otolaryngology - Head and Neck Surgery, Kumpulan Perubatan Johor Tawakkal KL Specialist Hospital, Kuala Lumpur, MYS; 3 Otolaryngology - Head and Neck Surgery, Queen Elizabeth Hospital, Kota Kinabalu, MYS

**Keywords:** eustachian tube balloon dilation, ventilatory tube insertion, myringotomy, facial canal wall dehiscence, facial baroparesis

## Abstract

Recurrent facial baroparesis is a rare condition that is mostly observed in individuals who have been exposed to barotraumatic conditions, particularly scuba divers and air travelers. We present a case of an unusual bilateral alternating recurrent facial nerve palsy and its successful treatment. A 34-year-old airline stewardess presented with a seven-month history of recurrent bilateral alternating facial nerve palsy that occurred exclusively during airline takeoffs. A clinical diagnosis of facial baroparesis was made. The temporal bone’s high-resolution CT (HRCT) scan revealed a bilateral tympanic segment of the facial canal wall dehiscence. Conservative treatment with oral antihistamines and nasal decongestants proved ineffective in treating this unusual condition. The patient then underwent bilateral Eustachian tube dilatation as well as bilateral myringotomy and grommet insertion. Post-treatment, she became asymptomatic despite multiple re-exposure to high-altitude travel. With our successful reported case of this uncommon recurrent condition, Eustachian tube dilatation as well as myringotomy and grommet insertion could potentially become the standard approach to treatment.

## Introduction

Recurrent facial baroparesis is a rare condition that mainly affects scuba divers and air travelers. This occurrence is presumed to be the result of increased middle-ear pressure with concurrent Eustachian tube dysfunction in the setting of a dehiscent facial canal wall. The pathophysiology of recurrent facial baroparesis is not fully understood, but the dehiscent facial canal wall in affected patients may expose the nerve to increased middle-ear pressure, causing transient ischemic neuropraxia [1-5]. We present a case of an airline stewardess with recurrent facial baroparesis, which was successfully treated with bilateral Eustachian tube balloon dilation, followed by bilateral myringotomy and Shepard grommet insertion.

Based on our case report, prompt Eustachian tube dilatation as well as myringotomy and grommet insertions may be treatment options for individuals suffering from this rare recurrent condition. According to our literature review, this is the first case of successfully treated facial baroparesis reported in Malaysia.

## Case presentation

**Figure 1:**
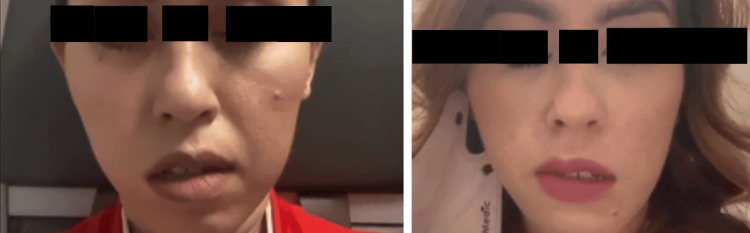
Two occasions when the patient developed bilateral alternating facial nerve palsy minutes after the plane took off.

A 34-year-old airline stewardess with no known medical conditions presented with recurrent bilateral alternating facial nerve palsy for the past seven months. As a stewardess, she frequently flew for work and had never experienced facial palsy before. However, in the past seven months, she experienced four episodes in total. During each transient occasion, she experienced unilateral facial asymmetry, inability to fully close one eye, loss of one-sided forehead wrinkling, and drooping of the corner of her lips. Three of the four episodes affected the right side of her face, while one episode affected the left (Figure 1). Symptoms occurred almost exclusively when the plane took off, yet spontaneously and completely resolved after 45 to 60 minutes. Each episode was preceded by her inability to equalize middle-ear pressures whenever she sensed aural fullness, which was accompanied by occasional nasal congestion. She denied having concurrent ear infections, hearing loss, vertigo, or tinnitus. During the transient facial nerve palsy, she had no headache, nausea, vomiting, or body weakness. These recurring episodes had imposed a significant physical and emotional burden on her as they would occur when she was on duty.

She was first assessed in the emergency and trauma department, where a workup was performed to rule out a central cause of facial palsy. A CT brain scan showed no intracranial abnormalities.

**Figure 2:**
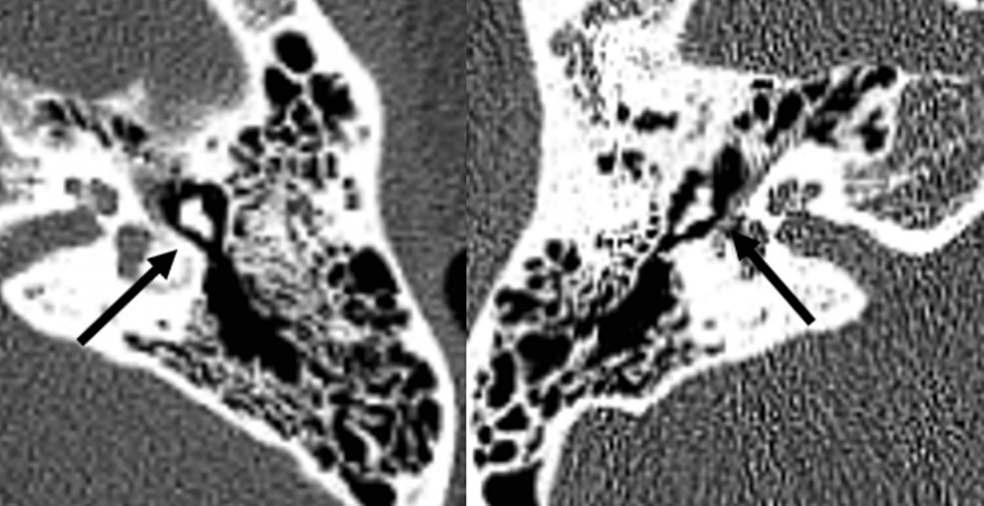
Axial thin-cut bone window CT scan of the temporal bone showing a bilateral tympanic segment of facial canal wall dehiscence (black arrows).

She was subsequently referred to our otorhinolaryngology clinic. At the time of the review, she was completely asymptomatic. Her bilateral facial nerves were intact. Other cranial nerve examination findings were unremarkable. On otoscopic examination, her bilateral tympanic membranes appeared retracted with a Sade grade four. A nasal endoscopy also revealed mild bilateral inferior turbinate hypertrophy with bulky Eustachian tube orifices. Moreover, pure tone audiometry indicated mild left sensorineural hearing loss at 250 Hz and 500 Hz with normal right hearing sensitivity. Tympanometry results found type AD in the right ear and type A in the left. The HRCT temporal scan revealed a bilateral tympanic segment of facial canal dehiscence (Figure 2).

We prescribed her an oral antihistamine and nasal decongestant. She took them 30 minutes prior to taking off. Despite compliance with both medications for two months, she reported a similar recurrence of facial palsy. Thus, we proceeded with bilateral Eustachian tube dilatation followed by bilateral myringotomy and Shepard grommet insertion one week later.

During the subsequent eight-month follow-up, she observed no more occurrences of facial palsy while on air and resumed her work as a flight attendant without any complications.

## Discussion

The exact cause of facial baroparesis is not fully understood, but it is thought to involve facial neuropraxia triggered by compression of the facial nerve in the presence of heightened middle-ear pressure and a dehiscent facial canal [1-5]. Commercial aircraft typically operate at cruising altitudes between 30,000 and 40,000 feet, with cabin pressurization ranging from 12 psi to 11 psi, equivalent to an altitude of 5000 to 8000 feet [1]. This pressurization leads to the expansion of gas in enclosed body spaces, such as the sinuses and middle ear, causing non-physiological gas collections. As an aircraft ascends, the atmospheric pressure decreases, causing the gas in the middle ear to expand according to Boyle's law. This creates a potential pressure difference across the tympanic membrane, typically equalized by the passive opening of the Eustachian tube, allowing pressure to vent through the nasopharynx. Dysfunctional Eustachian tube, resulting from conditions narrowing the lumen due to edema, increased mucous viscosity, or impaired normal opening, can lead to inadequate pressure equalization. If the Eustachian tube fails to vent air pressure from the middle ear to the nasopharynx during ascent, excessive middle-ear pressure may build up, potentially transmitting pressure to the facial nerve. The prevailing hypothesis in the literature attributes facial baroparesis to ischemic neuropraxia, based on the rapid onset and resolution of symptoms. It is theorized that excessive pressure transmitted through a dehiscence in the facial nerve canal reduces blood flow to the vasa nervorum of the facial nerve, resulting in neuropraxia [5]. Our case supports the theory, as she presented with symptoms of a dysfunctional Eustachian tube; her CT scan also revealed that bilateral tympanic segments of her facial canal were dehiscent.

Compared to the existing literature, our patient reported a unique presentation. She reported bilateral alternating facial nerve palsy on different occasions. Out of the 23 reported cases of facial baroparesis in aviation, 21 reported unilateral facial nerve palsy and only one reported a bilateral occurrence on two separate occasions, with the remaining case not specifying which side was affected [5]. Medical interventions were administered to a total of 13 cases. Among them, a patient who delayed seeking medical attention received oral prednisolone, resulting in the restoration of normal facial motor function. Grommet insertion alone was performed in five patients, while one patient underwent grommet insertion along with septoplasty and turbinoplasty. Three patients were managed with nasal decongestant spray before flying, and one patient underwent Eustachian tube balloon dilatation. A patient with a known facial nerve schwannoma underwent a revision facial nerve decompression, leading to the normalization of symptoms. One patient declined grommet insertion when offered. The remaining 10 cases resolved spontaneously, requiring no medical intervention. All cases that received medical intervention reported successful outcomes [2-5]. In our case, we performed bilateral Eustachian tube balloon dilatation and myringotomy with grommet insertion. The combination of these two treatment modalities yielded positive outcomes, as our patient reported no recurrence of facial palsy despite re-exposure to high-altitude travel.

In our case, prior to our surgical intervention, she was on trial of oral antihistamine and nasal decongestant for two months. Despite her compliance, the medications were ineffective as she still had a recurrence of her facial nerve palsy while on duty. They were insufficient in resolving her issues due to the presence of recurrent otitis media with effusion. This recurrence may be attributed to the severity of her underlying Eustachian tube dysfunction, indicating that conservative management alone may not be adequate to address the issue. With the utilization of both surgical interventions, her underlying Eustachian tube dysfunction as well as recurrent otitis media effusion could be more effectively addressed than through conservative medical treatment alone.

She had been under our monthly follow-up for the first two months and subsequently two-month follow-up since she underwent both interventions. There was one unscheduled visit where she presented with flu symptoms and a suspected blockage of one of the grommets. It was later discovered that she had stopped taking her oral antihistamines medication. However, her flu symptoms subsequently resolved with the resumption of her tablet antihistamines and the application of antibiotic ear drops a week after that. She otherwise reported no recurrence of facial nerve palsy during the subsequent eight-month follow-up.

The intervention may have lasting consequences, such as the possibility of recurring ear infections, which in our reported case may also be contributed to by her non-compliance to medication. Nevertheless, by maintaining proper ear hygiene and adhering to oral antihistamines, the occurrence of these infections can be minimized. Regular follow-up appointments at our clinic will also be necessary.

## Conclusions

Further research into this rare condition is vital to prevent unnecessary workups for affected patients. There have been reports of divers and air travelers mistaking the condition for an air embolism or stroke, resulting in unwarranted emergency workup and non-targeted treatment. Further studies should also explore the pathophysiology and risk factors as well as compare treatment modalities. This will indirectly enhance the full understanding and treatment of this unusual condition. In facilities where Eustachian tube balloon dilatation is not easily accessible due to its high cost, initiating myringotomy and grommet insertion might be a more feasible option, given its widespread availability in most otolaryngology centers. Nonetheless, with the success of our reported case, the implementation of both bilateral Eustachian tube balloon dilatation along with myringotomy and grommet insertion could potentially impact the clinical practice guidelines in the management of this rare recurrent condition.
